# Pathological complete response after conversion therapy in unresectable hepatocellular carcinoma: a retrospective study

**DOI:** 10.1186/s12876-024-03298-5

**Published:** 2024-07-30

**Authors:** Junjun Jia, Chenyuan Ding, Mengjie Mao, Feng Gao, Zhou Shao, Min Zhang, Shusen Zheng

**Affiliations:** 1 Division of Hepatobiliary and Pancreatic Surgery, Department of Surgery, The First Affiliated Hospital, Zhejiang University School of Medicine, Hangzhou, Zhejiang Province 310003 China; 2 Xuanwu Hospital, Capital Medical University, Beijing, 100053 China; 3 Division of Operation Room, The First Affiliated Hospital, Zhejiang University School of Medicine, Hangzhou, Zhejiang Province 310003 China

**Keywords:** Conversion therapy, Hepatocellular carcinoma, Adjuvant therapy

## Abstract

**Background:**

Hepatocellular carcinoma is a highly lethal tumor worldwide, and China has a correspondingly high incidence and mortality rate. For patients with unresectable hepatocellular carcinoma, the prognosis is often poor. The objective of this retrospective study was to investigate the effects of conversion therapies on these patients.

**Methods:**

The study included patients between the ages of 18 and 75 who were initially diagnosed with unresectable hepatocellular carcinoma and received conversion therapy. After completing surgery, the patients underwent pathological diagnosis, which showed complete necrosis. The study was conducted retrospectively at the First Affiliated Hospital, Zhejiang University School of Medicine, from January 2019 to December 2021. The main objectives of the study were to evaluate the overall survival and recurrence-free survival.

**Results:**

A total of 60 patients who met the inclusion criteria were enrolled. The median age of the patients was 56.6 ± 9.5 years, and 85% of them were male. The one-year overall survival rate (OS) was 98.3%, and the three-year OS was 95.6%. The one-year recurrence-free survival rate (RFS) was 81.1%, and the three-year RFS was 71.4%. In subgroup analysis, there was no statistically significant difference in RFS between patients with BCLC stages 0-A and BCLC stages B-C (*p* = 0.296). Additionally, there was no statistically significant difference in RFS between patients who received postoperative new adjuvant therapy and those who did not (*p* = 0.324).

**Conclusions:**

Conversion therapy followed by surgical resection could be a promising treatment for patients with initially unresectable hepatocellular carcinoma, and the prognosis is good with a pathological complete response.

## Introduction

Hepatocellular carcinoma (HCC) is the third leading cause of death in the world. With a 5-year survival of 18%, HCC is the second most lethal tumor in China by 2020. Only 5–10% of patients are ideal for resections, and the recurrence rate is still as high as 70% in five years postoperatively [1–4]. Unresectable hepatocellular carcinoma (uHCC) represents a heterogeneous group of patients who are deemed unresectable due to tumor characteristics such as metastatic disease or invasion into the vasculature. Despite efforts to enhance screening accessibility, a significant number of patients in China are diagnosed with uHCC, which leads to a high incidence and mortality. Therefore, it is crucial to consider conversion therapy for these patients [5, 6].

At present, conversion therapy can be divided into three main approaches. Firstly, for patients with poor liver function, efforts can be made to improve their liver function. Secondly, it is important to increase the remaining liver volume to meet the surgical requirements. Thirdly, the size of the tumor, the number of tumors, or the presence of metastasis could be reduced to down-stage HCC. Specific treatments are used, for example, transcatheter arterial embolization chemotherapy (TACE), stereotactic body radiotherapy (SBRT), portal vein embolization (PVE), systemic therapy like tyrosine kinase inhibitors (TKI), immune checkpoint inhibitors (ICI) and other methods to make the tumors resectable [7]. PVE is typically utilized to enhance the remaining liver volume. TACE and TKI/ICI may also be administered before the operation to reduce the tumor size or number. Surgical resection was performed after multi-disciplinary treatment. After surgery, some patients will receive TACE, TKI or ICI, which is defined as adjuvant therapy, to reduce the probability of recurrence and metastasis and improve survival time.

With the progress of systemic therapy, such as TKI/ICI, the conversion therapy of uHCC has become a hot spot in current research. This new therapeutic option offers hope to patients who were previously unable to undergo surgery. It has the potential to increase their life expectancy, particularly in the case of HCC which is becoming more widespread. However, studies about this topic are often carried out in single centers, and single-arm, and the sample size is relatively small [8]. In addition, domestic and foreign studies have targeted different ethnic patients, and whether the results are suitable for China, in which people with HBV-related HCC, is still unknown [9]. To validate the efficacy of conversion treatment for patients with uHCC, it is essential to conduct studies with a larger sample size. This article aims to summarize the treatment experiences of 60 patients who underwent conversion therapy and achieved pathological complete responses. Hoping to gain insights into the significance of conversion therapy in terms of disease recurrence and survival rates for patients with uHCC.

## Methods

### Study design and participants

Sixty patients with pathological complete response after conversion therapy at The First Affiliated Hospital, Zhejiang University from January 2019 to December 2021 were included.

Inclusion criteria were: [1] Initially tumor unresectable according to the multidisciplinary team [2] Patient who received surgery after conversion therapy [3] Pathological confirmed complete response [4] Patients aged 18–75 years with liver function of Child A.

The primary endpoints were overall survival (OS) and recurrence-free survival (RFS). The study was conducted according to the Declaration of Helsinki and the Good Clinical Practice standards, and approved by the Ethics Committee of the First Affiliated Hospital, Zhejiang University School of Medicine. All patients gave their informed consent prior to inclusion in the study.

Conversion therapy includes systemic therapy such as TKI, ICI, TACE, SBRT, PVE and so on. Pathological complete response is defined as the absence of any viable tumor cells after complete evaluation of the resected specimen, including all regional lymph nodes, emboli, and distant metastases sampled, and review of all slides [10].

Besides, postoperative adjuvant therapy was evaluated on postoperative outcomes. Postoperative adjuvant therapies include TACE, TKI or ICI, a combination of TACE, TKI and ICI.

### Procedures and treatment

The general procedure of TACE is to puncture the femoral artery or the radial artery, insert the guide wire into the hepatic artery, inject chemotherapy drugs into the hepatic artery branch, and embolize the vessel. Chemotherapy drugs usually mix iodized oil with cisplatin (1 mg/mL), other options are doxorubicin, carboplatin, oxaliplatin, mitomycin, fluorouracil et al. Surgical removal is evaluated 4–6 weeks after TACE. SBRT uses a stereotactic radiotherapy technique to ablate the tumor directly. The frequency of radiotherapy was once a day and 5 times a week. The course is usually 3–5 weeks. Surgical removal is evaluated five weeks after SBRT. PVE is mainly performed by ultrasound-guided percutaneous hepatic portal vein puncture, which embolizes the portal vein which needs to be excised, so that the non-tumor invaded liver lobe can proliferate. Surgical removal is performed 4–6 weeks after PVE. TKI commonly used is Sorafenib and Renvastinib. ICI generally includes Pabolizumab, Toripalimab, Sindillizumab, Tirellizumab, etc. Surgical removal is performed 1–2 weeks after TKI, while 4–6 weeks after ICI [10].

### Statistical analysis

Statistical analyses were conducted using SPSS 17.0 for Windows (SPSS, Inc., Chicago, IL, USA). The survival rate of patients was analyzed by the Kaplan–Meier method using GraphPad Prism 8. Statistical significance was set at *P* < 0.05.

## Results

### Demographic characteristics

For the study, a total of 60 patients were eligible. All the patients received preoperative TACE while 39 (60%) underwent systemic treatment such as TKI or ICI. 8 (13.3%) patients received PVE, and another 8(13.3%) received SBRT. The liver function of all these patients belonged to Child A. 42 (70%) patients received radical resection after conversion therapy within 2 months. All of them underwent resection surgery and obtained pathological results. Of these patients, 12 received postoperative adjuvant therapy and 48 did not. The demographics and baseline characteristics have been summarized in Table 1.

**Table 1:** Baseline data of patients included in the study

Variable	*N* = 60		*N* = 60
Age (years)	56.6 ± 9.5	Preoperative therapy, n (%)	
Gender, n (%)		TACE only	21(35%)
Male	51(95%)	TACE + TKI/ICI	39(65%)
Female	9(15%)	Preoperative PVE, n (%)	8(13.3%)
HCC etiology, n (%)		Preoperative SBRT, n (%)	8(13.3%)
HBV	48(80%)	Postoperative adjuvant therapy, n (%)	
HCV	1(1.7%)	TACE	4(6.7%)
Others	11(18.3%)	TKI/ICI	4(6.7%)
AFP, n(%)		TACE + TKI + ICI	4(6.7%)
< 400 ng/mL	38(63.3%)	No	48(80%)
≥ 400 ng/mL	22(36.7%)	Tumor lesion, n (%)	
BCLC, n (%)		single	42(70%)
Stage 0-A	31(51.7%)	multi	18(30%)
Stage B	5(8.3%)	Tumor cumulative diameter, n (%)	
Stage C	24(40%)	<5 cm	19(31.7%)
Tumor size, n(%)		>5 cm	41(68.3%)
<2 cm	3(6%)	Conversion time, n (%)	
2–5 cm	15(25%)	<2months	42(70%)
5–10 cm	33(55%)	2-4months	11(18.4%)
>10 cm	9(15%)	>4months	7(11.7%)
PVTT, n(%)			
Yes	18(75%)		
No	6(25%)		

*Note*:Tumor size refers to a single tumor diameter, and tumor cumulative diameter refers to the total diameter accumulated by multiple tumors. BCLC: Barcelona Clinic Liver Cancer. PVTT, n(%) means the percentage of PVTT in BCLC C patients

### Overall survival and recurrence-free survival

**Fig. 1:**
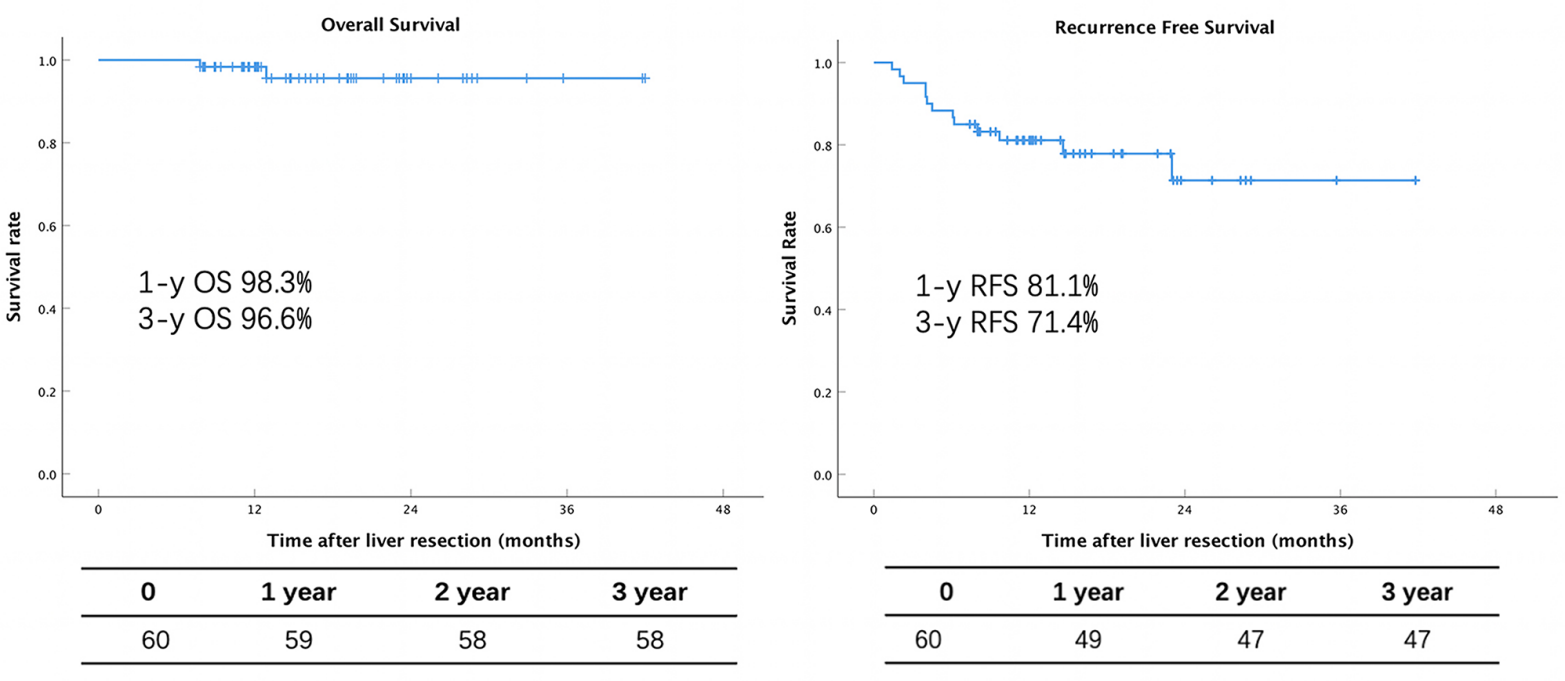
The OS and RFS after liver resection

The figures presented in Fig. 1 illustrate the outcomes of the OS and RFS. The 1-year OS rate was f 98.3%, while the 3-year OS rate was 96.6%. However, there were two deaths during follow-up. The cause of death was that one patient died 7.8 months after surgery due to severe infection caused by repeated biliary fistula, while the other patient died 12.9 months after surgery because this patient suffered bile duct obstruction and underwent a second operation, and finally died of repeated infection and liver failure.

In our cohort, the RFS rate at 1-year was 81.1%, while the 3-year RFS rate was 71.4%. Recurrence was observed in 13 cases during follow-up, with most cases occurring within nine months post-liver resection. Among these patients, two underwent a second operation, five received radiofrequency ablation (RFA), and two received SBRT. The remaining four patients received TACE combined with TKI.

**Fig. 2:**
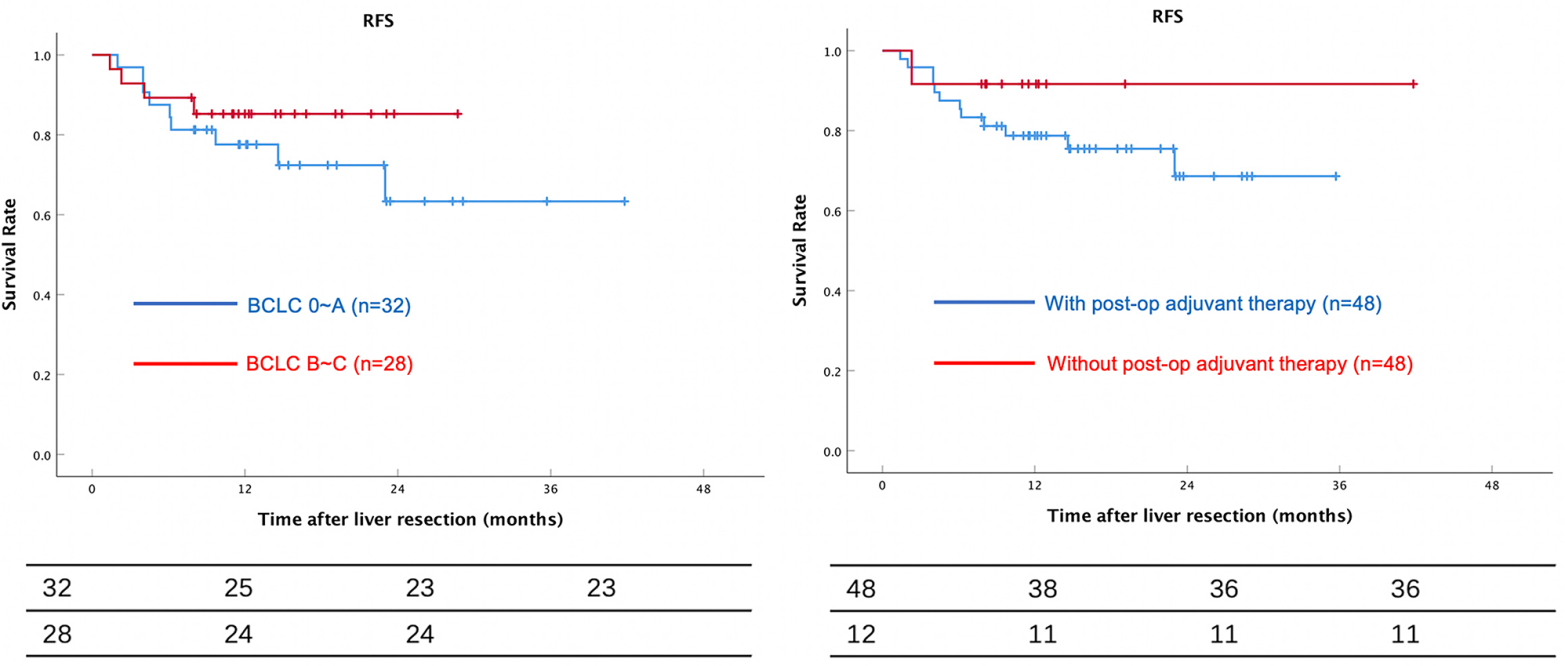
RFS according to BCLC and RFS with or without postoperative adjuvant therapy

Patients were divided into two groups according to their BCLC stages. 32 patients were BCLC 0-A, and 28 patients were BCLC B-C. The results showed patients with BCLC 0-A tend to have a better survival rate compared to BCLC B-C. However, the RFS data between the two groups had no statistical significance (Fig. 2, *P* = 0.294).

48 patients have no postoperative adjuvant therapy, and 12 patients have TACE(*n* = 4), TKI/ICI(*n* = 4), or TACE + TKI/ICI(*n* = 4) respectively after liver resection. Patients with postoperative adjuvant therapy had a similar RFS and there was no statistical significance either (Fig. 2, *P* = 0.394).

## Discussion

There are 60 patients included in the study, most were middle-aged and 80% of them had hepatitis B. The most used strategies for conversion therapy were TACE or TACE combined with ICI and/or TKI. Conversion therapy can quickly shrink tumors over the short term (70% within 2 months in our cohort), allowing for surgical resection and improved outcomes. However, postoperative adjuvant therapy did not have a significant impact on prognosis. With a pathological complete response, conversion therapy may lead to relatively high rates of overall survival (OS) and recurrence-free survival (RFS).

Conversion therapy is a combination of systemic and locoregional treatments for uHCC. It offers a chance for selected patients to undergo surgical resection by adequately downstaging the tumor [10]. The history of conversion therapy for liver cancer can be traced back to the 1970s [11, 12]. In the 1990s, many centers reported evidence of downstaging of HCC after therapy followed by resection [13–15]. A 2022 study found that sequential therapy with pembrolizumab-lenvatinib-TACE may have promising anti-tumor activity, an acceptable conversion rate, and a well-characterized safety profile in patients with uHCC who were initially PD-L1 positive [16]. Sequential TACE and SBRT followed by ICI therapy were considered as a viable conversion therapy for curative treatment [17]. . Meanwhile, other studies have shown that ALPPS may be significantly more effective than TACE or TACE + PVE, and the OS was longer [18]. However, conversion therapy is still a new approach to the treatment of uHCC and its practice and treatment protocols are still being developed.

Studies showed that MDT might induce a more profound and durable tumor response in patients with uHCC [17]. Surgery remains the core treatment for patients with HCC to achieve the best survival benefit. With advances in TKI as well as SBRT, multimodal conversion therapies are being explored to improve the proportion of inoperable HCCs, thereby improving the long-term prognosis for patients with advanced HCC [19]. However, there is no evidence to support the comparison between surgical and non-surgical treatment of uHCC with complete pathological response in long-term survival. To prove the complete pathological response, a complete assessment of the resected specimens, including all sampled regional lymph nodes, tumor thrombi, and distant metastases, and after reviewing all sections, no viable tumor cells were found is needed [10]. Without surgical treatment to remove all primary and metastatic lesions, there is no guarantee that whether the patient will achieve a complete pathological response.

Although resection is the primary goal of conversion therapy, it is not the goal of treating patients with uHCC. MDT is needed when implementing conversion therapy and determining treatment strategies after conversion therapy. Maximizing patient benefits should be the sole goal of collaboration.

Post-operative adjuvant therapy aims to reduce the risk of cancer recurrence. However, there is no high-level evidence for post-operative treatment after R0 resection, which means that the tumor is completely removed surgically and the margin is negative when viewed under the microscope. The choice of postoperative treatment regimen should be deliberated considering effectiveness and safety. Our adjuvant treatment included TKI and/or ICI, sometimes combined with TACE. And postoperative adjuvant therapy usually last for more than 6 months with a close observation every 2–3 months. If there are no signs of tumor recurrence and metastasis in two consecutive imaging examinations and the tumor markers (AFP, PIVKA-II), the adjuvant therapy can be suspended [10].

There were some limitations in our study. Firstly, it was a single-arm study, and the sample size was relatively small. Secondly, follow-up time is relatively short, which may not be very convincing to results. Thirdly, the effectiveness of different regimens of conversion therapy cannot be compared in detail. These are also the directions for our next research.

## Conclusion

Conversion therapy followed by surgical resection could be a promising treatment for patients with initial uHCC, and the prognosis is good with pathological complete response. Nonetheless, due to the retrospective nature of the current study, further investigation such as randomized trials, is required in the overall uHCC management landscape.

## Data Availability

The datasets used and/or analyzed during the current study are available from the corresponding author on reasonable request.

## Abbreviations

TACE: Transarterial Chemoembolization
uHCC: unresectable Hepatocellular Carcinoma
TKI: Tyrosine Kinase Inhibitors
ICI: Immune Checkpoint Inhibitors
PVE: Portal Vein Embolization
SBRT: Stereotactic Body Radiation Therapy
HBV: Hepatitis B Virus
HCV: Hepatitis C Virus
OS: Overall Survival
RFS: Recurrence-Free Survival
RFA: Radiofrequency Ablation
BCLC: Barcelona Clinic Liver Cancer
PVTT: Portal Vein Tumor Thrombus
